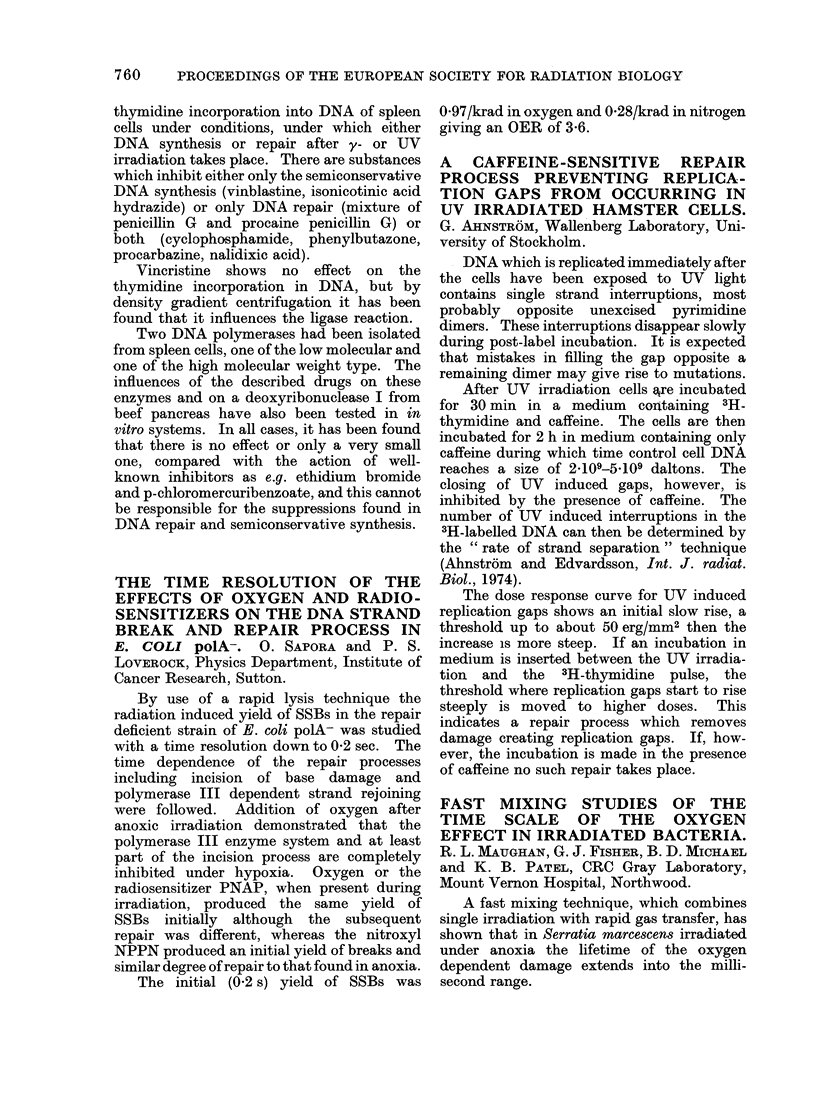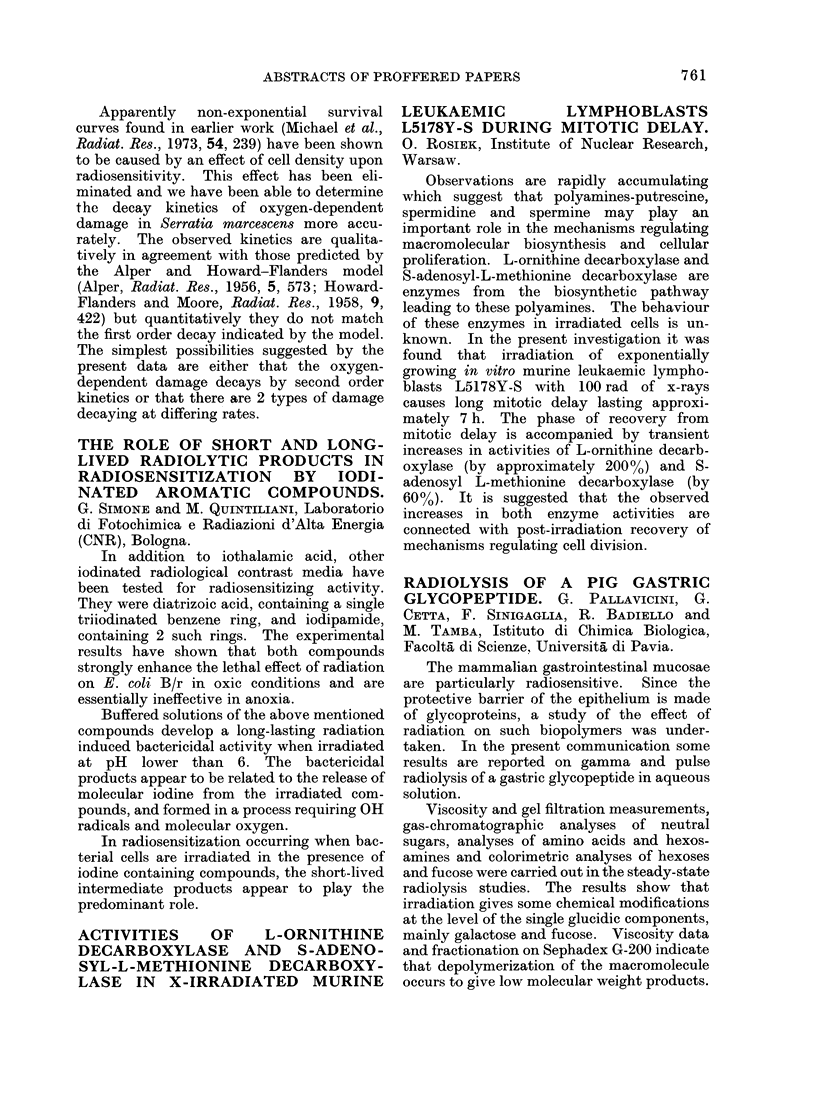# Proceedings: Fast mixing studies on the time scale of the oxygen effect in irradiated bacteria.

**DOI:** 10.1038/bjc.1975.321

**Published:** 1975-12

**Authors:** R. L. Maughan, G. J. Fisher, B. D. Michael, K. B. Patel


### FAST MIXING STUDIES OF THE TIME SCALE OF THE OXYGEN EFFECT IN IRRADIATED BACTERIA.

R. L. MAUGHAN, G. J. FISHER, B. D. MICHAEL and K. B. PATEL, CRC Gray Laboratory, Mount Vernon Hospital, Northwood.

A fast mixing technique, which combines single irradiation with rapid gas transfer, has shown that in *Serratia marcescens* irradiated under anoxia the lifetime of the oxygen dependent damage extends into the millisecond range.

Apparently non-exponential survival curves found in earlier work (Michael *et al.*, *Radiat. Res.*, 1973, **54**, 239) have been shown to be caused by an effect of cell density upon radiosensitivity. This effect has been eliminated and we have been able to determine the decay kinetics of oxygen-dependent damage in *Serratia marcescens* more accurately. The observed kinetics are qualitatively in agreement with those predicted by the Alper and Howard–Flanders model (Alper, *Radiat. Res.*, 1956, **5**, 573; Howard-Flanders and Moore, *Radiat. Res.*, 1958, **9**, 422) but quantitatively they do not match the first order decay indicated by the model. The simplest possibilities suggested by the present data are either that the oxygen-dependent damage decays by second order kinetics or that there are 2 types of damage decaying at differing rates.